# Fluorescence lifetime imaging with distance and ranging using a miniaturised SPAD system

**DOI:** 10.1038/s41598-024-63409-w

**Published:** 2024-06-10

**Authors:** Andrew B. Matheson, Charlotte Hopkinson, Michael G. Tanner, Robert K. Henderson

**Affiliations:** 1 School of Engineering, Institute for Integrated Micro and Nano Systems, University of Edinburgh, Edinburgh, EH9 3FF UK; 2 School of Engineering and Physical Sciences, Institute of Photonics and Quantum Sciences, Heriot-Watt University, Edinburgh, EH14 4AS UK

**Keywords:** Optical sensors, Imaging and sensing, Endoscopy, Three-dimensional imaging

## Abstract

In this work we demonstrate a miniaturised imaging system based around a time-gated SPAD array operating in a “chip-on-tip” manner. Two versions of the system are demonstrated, each measuring 23 mm × 23 mm × 28 mm with differing fields of view and working distances. Initial tests demonstrate contrast between materials in widefield fluorescence imaging (WFLIm) mode, with frame rates of > 2 Hz achievable. Following this, WFLIm images of autofluorescence in ovine lung tissue are obtained at frame rates of ~ 1 Hz. Finally, the ability of the second system to perform simultaneous WFLIm and time of flight (aka Flourescence Lifetime Imaging Distance and Ranging, FLImDAR) is also tested. This shows that the system is capable of 4 mm resolution of object separation when tested on 3D printed samples. It is further demonstrated as being able to perform scene reconstruction on autofluorescent lung tissue. This system is, to date, the smallest chip on tip WFLIm system published, and is the first demonstration of the FLImDAR technique in a compact, portable system.

## Introduction

Silicon Single Photon Avalanche Diode (SPAD) arrays offer excellent sensitivity and low dark count rates^1–3^. Importantly, the electronics to allow for time gating, time correlated single photon counting (TCSPC), and some data processing, can be integrated on the pixel level^3^. This makes them particularly suitable for applications such as Fluorescence Lifetime Imaging Microscopy (FLIM), and Widefield Fluorescence Lifetime Imaging (WFLIm)^4–6^. WFLIm is the imaging modality where the fluorescence decay from a macro-scale object is captured with fine enough time resolution that the fluorescent lifetime can be calculated^7–11^. This can provide additional contrast not available from fluorescence intensity alone, highlighting variations in material properties or composition. It should be noted that SPAD’s are not the only means for performing fluorescent lifetime imaging, and other technologies and techniques show much promise^12,13^.

Endocam is a 120 × 128 SPAD array with a < 2 mm^2^ footprint developed by this group. It has so far been employed for such diverse applications as explosive vapour sensing^6^, plant health^4^, and the differentiation between regions in stained human lung tissue^14^. The most significant difference between Endocam and other time-gated SPAD arrays is its small form factor and ability to operate at a distance from its control board over a five wire interface. Although other miniaturised chip-on-tip cameras for endoscopic applications exist, as summarised in Ref.^14^, this combination of very small form factor and time gated SPAD capabilities is unique. By pairing the Endocam sensor with a pulsed laser, multimode fibre and some basic off-the-shelf optics, this group previously demonstrated a handheld system with dimensions ~ 45 × 45 × 45 mm being used for WFLIm on a range of samples including mammalian tissue^4^.

Another common application of time gated SPAD arrays is in time-of-flight techniques such as LIDAR, where the time resolution is used to measure the round trip of photons scattered from objects, thus allowing for distances to be calculated. Recently, this group has demonstrated that WFLIm and ToF data may be extracted simultaneously from the same time resolved SPAD data to allow for 3D scene reconstruction and surface mapping of fluorescent objects^5,15^. This differs from most previous attempts to combine ToF and WFLIm, as these have tended to focus on locating exogenous fluorescent inclusions at depth in the sample^16–18^, and although fluorescence ToF/LIDAR is a well established technique, it has tended to look only at fluorescence intensity rather than lifetime^19–21^. We have summarised these techniques in Table S1. The composite technique, which we will refer to hereafter as FLImDAR (Fluorescence Lifetime Imaging with Distance And Ranging), has clear biomedical applications such as endoscopy, surgical guidance, and diagnostic imaging. As well as these applications in biomedical imaging, this combination of WFLIm and time of flight information could prove useful in fields such as surveying and agronomy where fluorescence lifetime may provide information on plant health and pathology^4,19,22^, or remote sensing in hazardous areas such as nuclear reactors. However, to date, FLImDAR has only been employed using TCSPC based systems. These are large benchtop systems with many data IO’s to facilitate the high bandwidth data transfer TCSPC requires.

In this work we demonstrate two systems based around the Endocam sensor, both with external dimensions of 23 × 23 × 28 mm. One system has been designed to have a working distance of 6 mm, to demonstrate the possible capabilities of the system for WFLIm in some in vivo application (e.g. dental imaging). The other has a larger field of view and a working distance of > 100 mm, suitable for uses such as surgical guidance, and allowing for the first demonstration of FLImDAR using a miniaturised handheld system. These modules constitute the smallest chip on tip fluorescence lifetime imaging systems published to date, building on previously published work, and represent an important staging post in the development of both FLImDAR and fluorescent lifetime endoscopy more generally.

## Materials and methods

### Imaging systems

The Endocam sensor is described in some detail elsewhere^4,14^. In short, it consists of 120 × 128 SPAD pixels, each with its own integrated photon counting electronics. A time gate can be set and moved in 0.379 ns increments and then applied to these pixels, either globally or with odd and even columns of pixels receiving different gates^14^ (see Fig. S1 in Supplementary Information for a schematic representation).

Time resolution can then be obtained by operating in sliding gate mode, whereby the temporal position of the gate is moved relative to an excitation laser pulse and a fluorescence decay curve reconstructed. Alternatively, the ratio of intensities obtained using two different time gate positions can give an approximate measure of lifetime using the rapid lifetime determination (RLD) method detailed in Eq. (1):

1 $$\tau =-\Delta t/{\text{ln}}({I}_{1}/{I}_{2} ),$$

where $\tau$ is the calculated lifetime, $\Delta t$ is the size of the time gate, and ${I}_{1}$ and ${I}_{2}$ are the intensities at the first and second time gate position respectively (see Fig. S2 in Supplementary Information). For RLD in this work, we exclusively use contiguous, non-overlapping gates of equal size. Time gating is, in general, less photon efficient than TCSPC, as only photons arriving during the open gate period are counted. However, it requires far less data bandwidth, making it an appealing option for the Endocam system.

**Figure 1:**
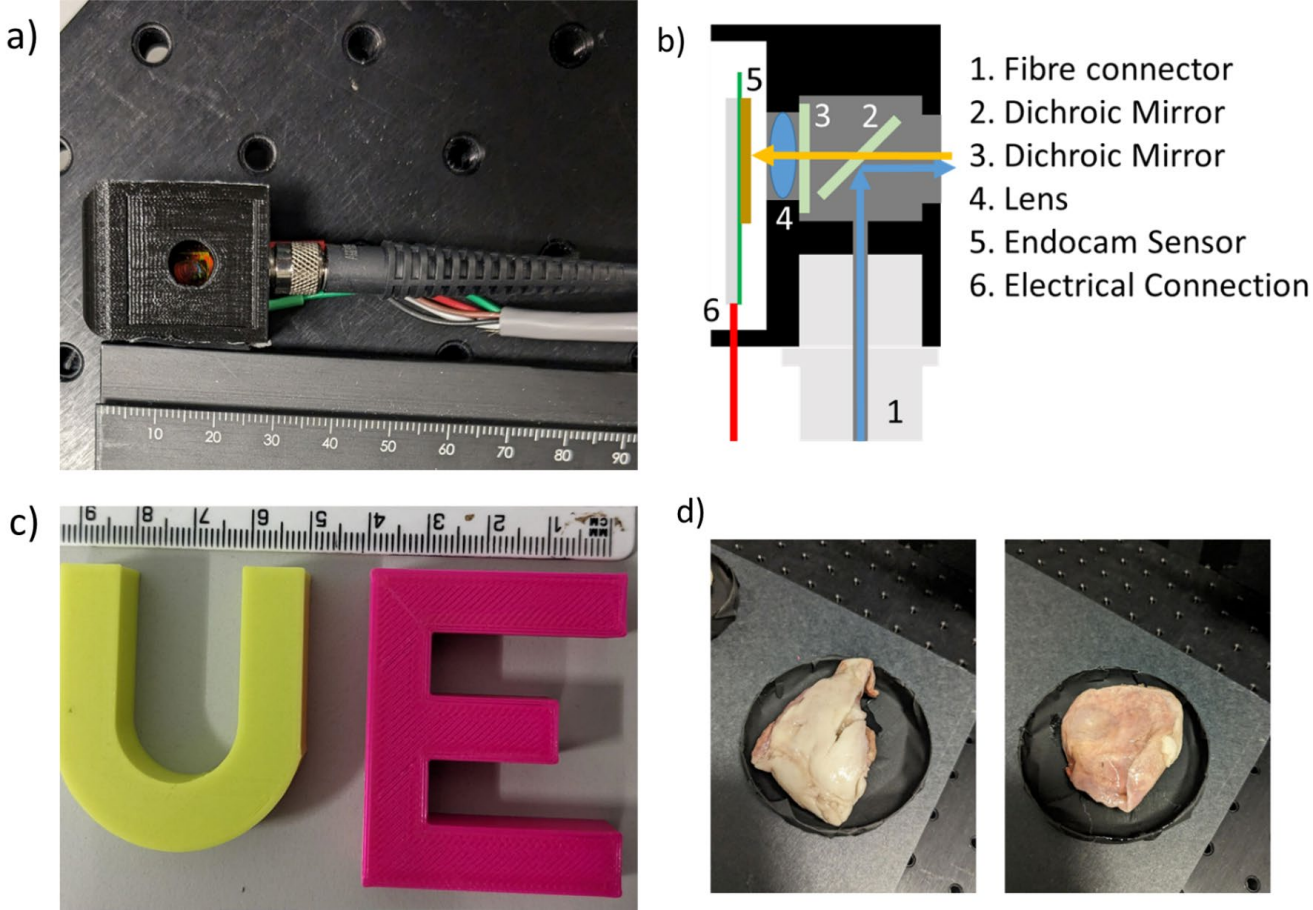
(**a**) Miniaturised Endocam system, (**b**) schematic of system, (**c**) photograph of 3D printed fluorescent test samples, (**d**) ovine lung tissue samples.

The Endocam sensor is designed to be able to operate on the distal end of an imaging system, and can be run over a ~ 1 m wired interface^4,23^. This can then be integrated into a small optical system, such as the one shown in Fig. 1a. A schematic of the system is included in Fig. 1b. To produce the miniaturised system presented in this work, a housing was 3D printed from poly-lactic acid (PLA) with external dimensions of 23 × 23 × 28 mm. This represents a factor of five reduction in volume compared to the first iteration of this system, which was small enough to be handheld but still too large for any in vivo applications^4^. Excitation light is provided by a Hamamatsu Picosecond Light Pulser PLP-10 laser diode head with λ = 483 nm, pulse-width = 80 ps which is coupled into multimode fibre (NA = 0.5, Thorlabs M124L02) giving ~ 0.3 mW of power at 20 MHz. Excitation light is then reflected at ~ 45° by a long pass dichroic mirror (Thorlabs DMLP550R, 550 nm cut on wavelength) out of the front aperture and onto the sample. The emitted fluorescence (orange arrow in Fig. 1b) passes through the aperture and the dichroic, and is further filtered by a second dichroic (also Thorlabs DMLP550R, placed normal to the beam path thus shifting the cut-on wavelength) to clean up any stray excitation light which is scattered within the housing. It is then focussed by a low-cost plastic aspheric lens (focal length = 3.32 mm, NA = 0.4, Thorlabs CAY033) and an image formed on the Endocam sensor. The sensor itself is mounted onto a custom PCB. A five wire connector sits on the back of the board, providing connections for data I/O, a 20 MHz timing clock, 2.8 V for the on chip micro-controller unit, 18.5 V for the SPAD’s, and a common ground connection.

As stated previously, two slightly different versions of the imaging head were produced with different distances between the lens and the sensor, allowing for one system to image at a working distance of ~ 6 mm and another to work at a distance of > 100 mm. This is summarised in Table 1.

**Table 1:** Summary of imaging heads.

	External dimensions (mm)	Working distance (mm)	Angular field of view
System A	23 × 23 × 28	> 100	~ 19°
System B	23 × 23 × 28	~ 6	~ 74°

### Sample preparation

Test samples for initial characterisation were prepared by 3D printing shapes from differing fluorescent filaments, and are shown in Fig. 1c. The green/yellow sample (colorFabb fluorescent green) consisted of fluorophores distributed within a PLA/PHA matrix with fluorophores embedded, and the pink (Real Filament fluorescent pink) consisted of fluorophores distributed throughout a matrix of just PLA. Although the precise fluorophores used in these filaments has not been shared by the manufacturer, they offer excellent fluorescent intensity and lifetime contrast and have been used widely for WFLIm characterisation previously^4,5,15^.

Ovine lung tissue samples are shown in Fig. 1d, samples from the same batch have recently been used to test FLImDAR capabilities in another instrument^5^. Ovine lungs were obtained from ewes destined for cull and euthanized under Schedule 1 of Animal (Scientific Procedures) Act 1986 and then frozen. These samples were provided to one of the authors as part of a previous study and no animals were handled by the authors. They were removed from the freezer and left to allow the surfaces to thaw before imaging experiments were carried out.

To avoid the influence of background light, all measurements were performed in a darkened room, this should replicate the conditions of using the system endoscopically in vivo.

## Results

### WFLIm

**Figure 2:**
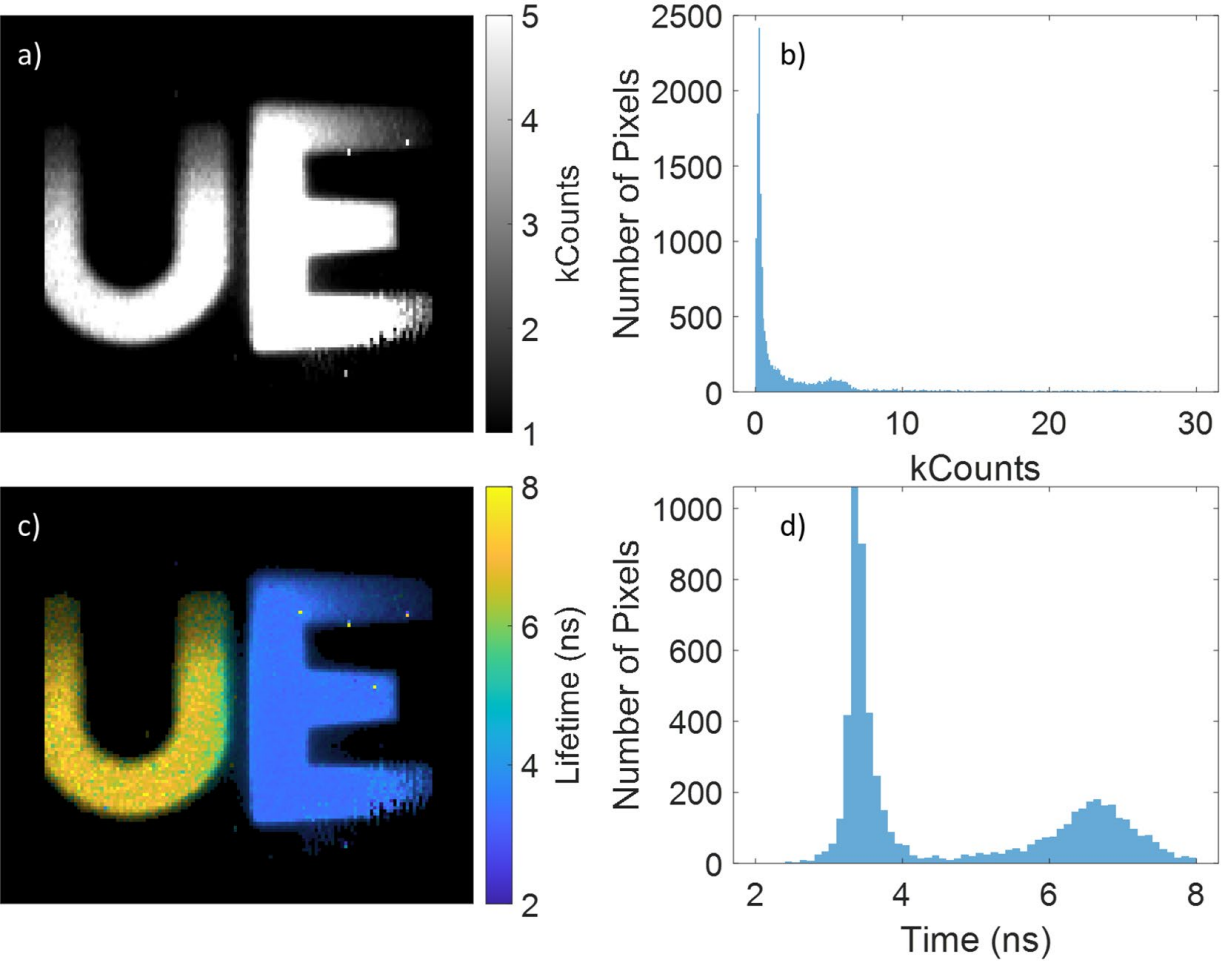
(**a**) An intensity image of 3D printed fluorescent samples, (**b**) the histogram of pixel intensity. (**c**) The WFLIm image obtained as part of the same image and (**d**) the associated lifetime histogram. Images taken as a gate sweep with 53 time steps each with an exposure of 3.3 s, using system A, with a total acquisition time of 10.4 min.

Initial tests for system A were carried out on the 3D printed samples using the sliding gate mode, as shown in Fig. 2. Both the letter U and E (for University of Edinburgh) are clearly visible in the intensity plot shown in Fig. 2a (maximum and minimum intensity level of image optimised to maximise contrast), however the histogram of the intensity image shown in Fig. 2b shows that there is poor differentiation between these materials. The WFLIm image shown in Fig. 2c (onto which an image alpha map has been applied to adjust brightness according to the intensity image) shows much better contrast, as evidenced by the histogram of the image shown in Fig. 2d, with two well defined peaks. The right-hand peak (associated with the U shape) is centred on 6.6 ns with a FWHM of ~ 1.1 ns. The left-hand peak (associated with the E) is centred on 3.3 ns with a FWHM of ~ 0.4 ns. The distribution is consistent with that obtained using a commercially available Horiba FLIMera FLIM camera looking at these sample, where the U has a peak at 6.5 ± 0.2 ns and the E peaks at 2.9 ± 0.1 ns.

**Figure 3:**
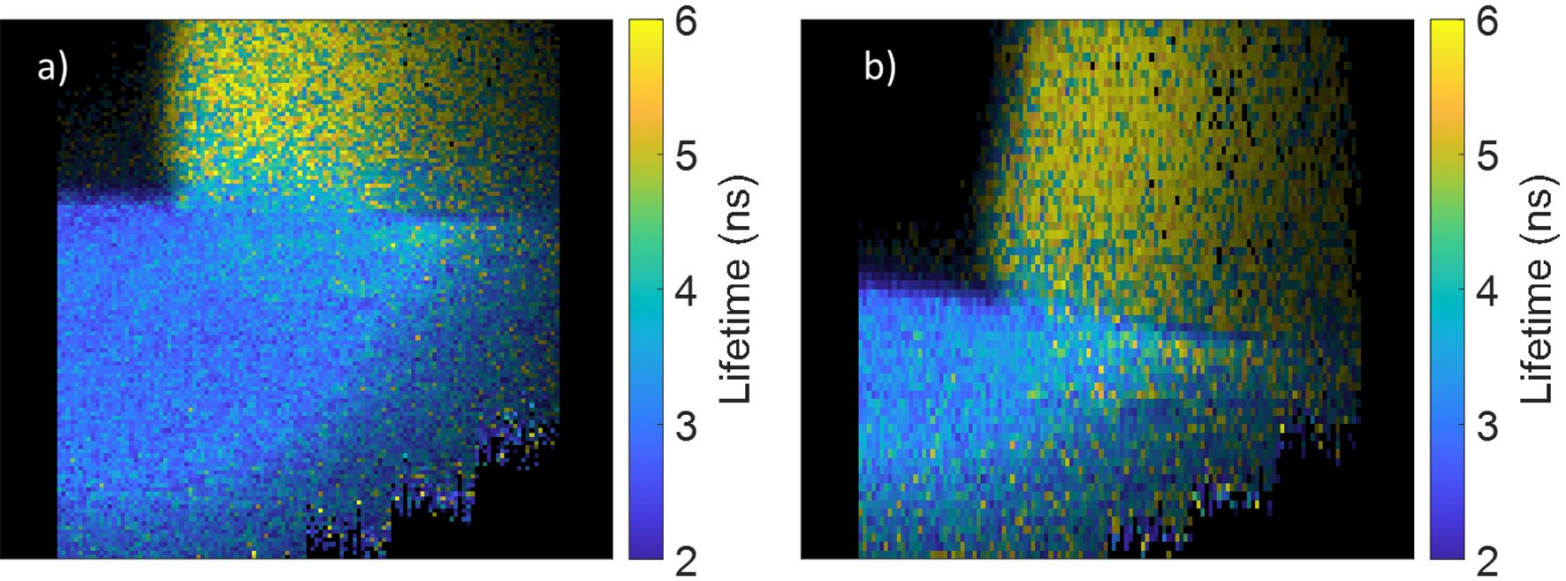
Still images from WFLIm videos of 3D printed test samples obtained with system B. Images were taken with (**a**) RLD with two contiguous gates using alternating frame gating (capturing two frames each of ~ 3 ms exposure at ~ 1.9 Hz) and (**b**) alternating column gating of pixels (capturing a single frame of ~ 3 ms exposure at 2.2 Hz). For both data sets ~ 1.9 ns gates were used, with the first gate positioned ~ 1.9 ns after the peak of the fluorescence.

The data shown in Fig. 2 were obtained operating in “gate-sweep” mode, resulting in long data acquisition times. For shorter acquisition times and faster frame rates, RLD mode is preferred. Figure 3a is of the fluorescent targets again, but now using system B, and has been generated by taking alternating frames with the time gate in the “I_1_” and “I_2_” position. This mode preserves the spatial resolution of the sensor, but results in some motion induced artefacts due to the gate positions not being captured simultaneously (see Video 1 in Supplementary Materials) when the objects are moved around the field of view by hand. Figure 3b, however, employs different gates within the same image—odd numbered columns of pixels use one gate whereas even columns of pixels use the other. As can be seen, the spatial resolution of this image is halved along the y axis of this image, but the image can be obtained more quickly allowing us to achieve frame rates of > 2 Hz. It should be noted that halving the spatial resolution in this manner does not result in a doubling in the frame rate, as the capture rate is partially limited by the communication overhead. However, frame rate is not the primary benefit of this mode of operation, by capturing both time gates simultaneously motion induced artefacts in the lifetime images are greatly reduced (see Video 2 in Supplementary Materials, and Fig. S3 in SI which highlights these artefacts).

We have previously shown how WFLIm performed by the Endocam sensor can successfully differentiate between tissue types in ovine kidney^4^, and that FLIM performed using Endocam can be used to highlight contrast between regions of stained lung tissue^14^. Demonstrating that this ability to obtain WFLIm contrast is maintained even in this smaller system is thus of key importance.

**Figure 4:**
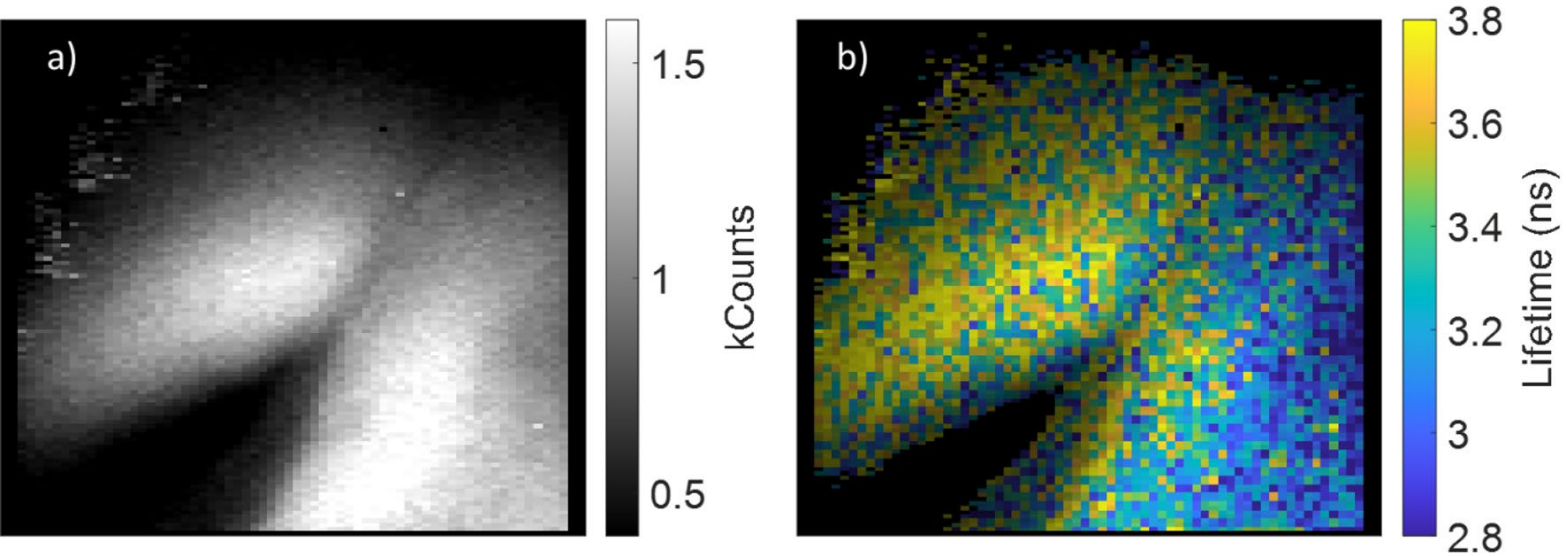
Intensity (**a**) and lifetime (**b**) images from a video of ovine lung tissue, obtained with system B operating in the RLD alternating gated pixel columns mode (2.3 ns contiguous gates are used and the first gate positioned ~ 1.1 ns after the peak of the fluorescence). Images taken at a frame rate of ~ 0.9 Hz (0.33 s exposure per image). To improve SNR the images are further spatially downsampled by a factor of two along the x and y axes.

Figure 4a shows intensity and Fig. 4b shows lifetime images obtained for ovine lung autofluorescence. To improve the signal to noise of these lifetime images, they have been spatially down-sampled by a factor of two prior to lifetime calculations being carried out. This results in a slightly more pixelated image, but as can be seen in Fig. 4b, there is clear contrast between the lifetimes of left and right-hand objects in the image when using the WFLIm modality, which are not evident from the intensity images alone (see Video 3 in Supplementary Materials for a full video). A system with the ability to differentiate between tissue types using only tissue autofluorescence, at frame rates close to 1 fps, in a form factor small enough for some in vivo uses could have myriad applications in biomedical imaging. It has already been demonstrated that this combination of sensor and laser when used as part of a benchtop microscope^14^, are capable of differentiating between tissues from human lung biopsy, this suggests possible applications for this system in the field of oncology or diagnostic imaging.

### FLImDAR

**Figure 5:**
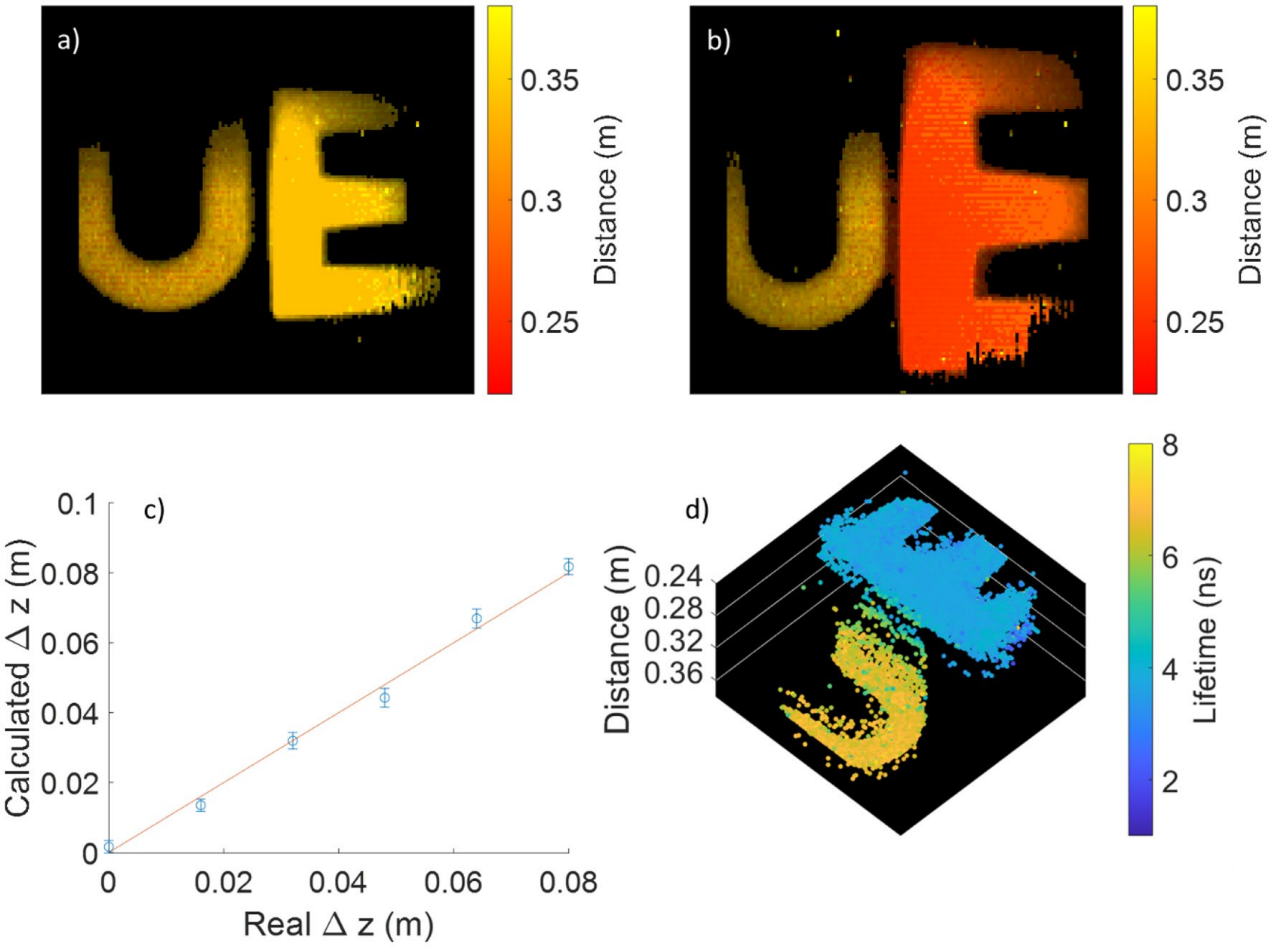
Distance images obtained for two 3D printed samples placed (**a**) at the same distance from the sensor and (**b**) with the “E” placed 80 mm closer than the “U”. (**c**) A plot to show the calculated object separation vs the real object separation, (**d**) a 3D point cloud of the two objects shown in (**b**), with the z axis showing the calculated distance and the colour of the points representing the lifetime. Images taken with system A. To generate these images, 53 bin positions with an exposure of 3.3 s per bin position were obtained, to give a total acquisition time of 10.4 min.

As mentioned previously, this group has recently demonstrated that time resolved SPAD sensors may be used to perform combined WFLIm and time of flight measurements to allow for simultaneous lifetime contrast and 3D scene reconstruction^5,15^. The means for doing so is by analysing the temporal position of the rising edge of the fluorescence signal for each pixel^5^. In Fig. 5 we demonstrate this capability using the miniaturised Endocam system, the first time this modality has been demonstrated using a handheld, mobile sensor in this manner, and also the first-time FLImDAR analysis has been carried out on time gated single photon counting rather than TCSPC data. As in the data in Fig. 2, gate sweeps were obtained for fifty-three time gate positions with an exposure of ~ 3.3 s per position, and analysis carried out. Figure 5a shows the U and E targets, with both objects the same distance from the sensor. As can be seen from the colour scale of the image, the system accurately discerns that both objects are a similar distance away (we are primarily interested in the relative distance of objects within the image, rather than absolute distance, and have not corrected for e.g. electronic delay in laser triggering, time of flight down the optical fibre etc.). As the E is brought forward, one sees that it becomes more red, an indication that it is closer to the sensor than the U. By splitting all the pixels on the left and the right of the image, applying an intensity threshold and then summing the counts, we may take the average position of each of the letters. We find that systematically, the right-hand object is calculated to be 11 mm further away from the camera than expected when compared to the left hand, stationary sample (see Fig. S4a). This systematic error is most likely due to the time for clock signals to traverse the width of the sensor. This offset can, however, be subtracted as a linear offset (see Fig. S4b for a comparison of data before and after this correction) and the object separation calculated. This is shown in Fig. 5c, as the E is moved through 6 different positions varying from 0 to 800 mm object separation. The error bars show the standard error in the calculated $\Delta z$ value. Taking the residual between the calculated spacing and the line showing the real spacing gives a maximum of just 4 mm. This resolution is very similar to that previously demonstrated by Stellinga et al. using a multimode optical fibre to perform time of flight 3D imaging^24^. Although the head of the system demonstrated here is significantly larger than the fibre tip used for their ultra-thin microendoscope, the optical configuration is considerably simpler on this chip on tip system, and with the additional benefit of providing fluorescence lifetime information, albeit with significantly longer acquisition times. Additionally, the imaging head of this FLImDAR system is just ~ 2% of the volume of the FLIMera TCSPC system previously used in Ref.^5^ (not even taking into account collection optics used in that work), and is the first demonstration of this technique in a miniaturised form factor.

The WFLIm and distance information may then be combined to generate the 3D point cloud shown in Fig. 5d, with distance represented on the z axis and point colour used to represent lifetime, the first time this sort of visualisation has been generated from FLImDAR data. Subsequent frames of this form may then be used to generated the videos shown in SI Video 4, where the E moves in time but preserves its lifetime.

**Figure 6:**
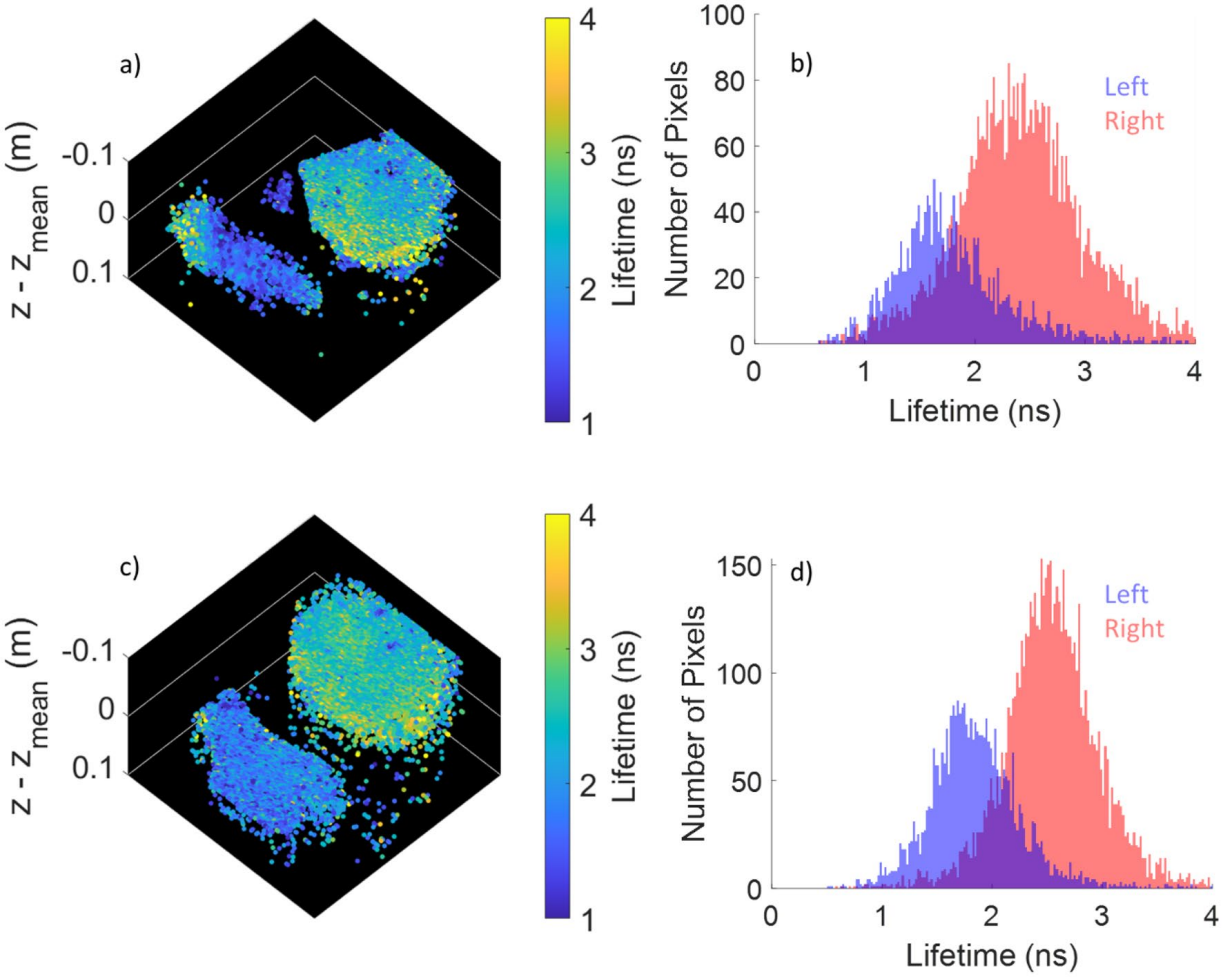
(**a**) FLImDAR 3D point clouds of $\text{z}-{\text{z}}_{\text{mean}}$ for two ovine lung tissue samples, (**b**) corresponding lifetime histogram for the left (blue) and right (pink) object shown in (**a,c,d**) as (**a,b**) but taken with the right hand object moved closer to the camera. Images taken with system A. To generate these images, 51 bin positions with an exposure of 3.3 s per bin position were obtained at a total acquisition time of 6.5 min per FLImDAR image.

Finally, FLImDAR and chip on tip imaging are brought together and applied to tissue autofluorescence, to produce the 3D point clouds for ovine lung tissue shown in Fig. 6. In panel a is the point cloud for two pieces of tissue kept equidistant to the camera. The mean value of z across the image has been subtracted to better highlight the topography. Panel b shows the associated lifetime histogram for the left (blue) and right (pink) piece. Both Fig. 6a and Fig. 6b show that the left-hand piece of tissue has a much shorter lifetime in general compared to the right-hand piece. Specifically, there is a line of material with a long lifetime around the bottom edge of the tissue which is associated with fat and cartilage (see Fig. S5). After one of the samples is moved closer to the camera, frame c is obtained. This clearly shows that the right-hand tissue is now closer to the camera and importantly, the colour of the objects in c and the associated lifetime histogram d show that the contrast is maintained as the distance has changed.

The acquisition time for these FLImDar images was ~ 390 s (exposure per bin position of 3.3 s over 51 bin positions, plus communications overheads), which is clearly not yet adequate for real diagnostic imaging applications. This was due to the large number of positions measured in the sweep of time gates, as well as the low autofluorescence intensity. For future experiments there is scope to measure over a narrower temporal window, and to increase the laser intensity and work at a shorter wavelength (where autofluorescence will be stronger) to increase the number of fluorescent photons.

Ultimately, the Endocam sensor used here is highly effective for chip on tip WFLIm, but was not specifically designed for the FLImDAR application. For instance, during the design process, the temporal resolution of Endocam was kept relatively course, but could be reduced in a subsequent system to improve spatial resolution in the depth axis^14^. For a future chip-on-tip FLImDAR system, a sensor capable of running over a wired interface with full on chip TCSPC timestamping (as used in previous benchtop FLImDAR systems^5,15^) and the latest SPAD technology^1,25,26^ would significantly increase the performance of SPAD-based endoscopic imagers. In particular, there have been rapid advances in shrinking SPAD pitch^25^ allowing around 10× more pixels in the same area as our present sensor’s pixel. The photon detection efficiency has increased by around 5× in the visible and considerably more in the NIR driven by LIDAR requirements. Although we have so far focused only on autofluorescence in the visible, this does raise the intriguing possibility of using NIR emitting stains and then focussing on this part of the spectrum instead. At the same time the dark count noise per unit area has been reduced by 10–50×^26^. We expect these developments if applied to future sensors would improve the photon budget to allow FLImDAR to be performed at video rates with milimetric precision. Despite this, the results shown in Fig. 6 still represent the first FLImDAR measurements performed with a chip on tip system, and given that neither has the sensor been specifically designed for this application, or exogenous stains used, are highly promising.

## Conclusions

In this work we have demonstrated the use of a miniaturised time-resolved imaging sensor, small enough to be considered for some in-vivo applications, and capable of providing contrast between different tissue types in ovine lungs using the fluorescence lifetime from autofluorescence at close to 1 frame per second. This significantly builds on the promise of previous work, and is the first chip-on-tip WFLIm system small enough for some in vivo applications. Additionally, this is the first demonstration of the new FLImDAR modality being deployed via a small handheld system. Although demonstrating slow acquisitions times, the ~ 4 mm spatial resolution demonstrated by the system when imaging bright samples, and the ability to obtain distance and lifetime simultaneously from ovine lung auto-fluorescence suggest that FLImDAR may be a viable modality in micro-endoscopic imaging.

### Supplementary Information

Supplementary Information.

Supplementary Video 1.

Supplementary Video 2.

Supplementary Video 3.

Supplementary Video 4.

## Data Availability

Data sets generated during the current study are available from the corresponding author on reasonable request.
