# Supplementary material for: Fluorescence lifetime imaging with distance and ranging using a miniaturised SPAD system
Source: Sci Rep. 2024 Jun 10;14:13285. doi: 10.1038/s41598-024-63409-w (PMC11164884; doi:10.1038/s41598-024-63409-w)
Supplement: Supplementary file 1 — Supplementary Information. [file 41598_2024_63409_MOESM1_ESM.docx]

Supplementary Information

# Captions for videos

Video 1 – WFLIm video of 3D printed test samples obtained with system B. Images were taken with RLD using alternating frame gating. Gates of size ~1.9 ns were used and an exposure of 3 ms per frame, giving a total acquisition rate of 1.9 Hz.

Video 2 - WFLIm video of 3D printed test samples obtained with system B. Images were taken with RLD with differing gates using alternating column gating of pixels. Gates of size ~1.9 ns were used and an exposure of 3 ms, giving a total acquisition rate of 2.2 Hz.

Video 3 – WFLIm video of ovine lung tissue, obtained using differing gates for alternating pixel columns and then spatially downsampling the image by a factor of two. Gates of size 2.3 ns were used and an exposure of 0.3 s, giving an acquisition rate of 0.9 Hz.

Video 4 – Sequence of 3D point clouds for fluorescent 3D printed targets in a range of different positions, with distance represented on the z axis and point colour used to represent lifetime. 53 gate positions were used and an exposure of 3.3 s per gate, giving a total acquisition time of 10.4 mins. Playback in this video is set to 1 fps.

Table S1 – Combined fluorescence and time of flight techniques

|  | **Target** | **Technology Used** | **WFLIm Capabilities** | **Time of Flight**  **Capabilities** |
| --- | --- | --- | --- | --- |
| **This Work** | 3D printed targets and unstained ovine tissue | Time gated SPAD array | Yes | Yes |
| **Hopkinson et al.**  **2023 *1*** | 3D printed targets and unstained ovine tissue | TCSPC enabled SPAD array | Yes | Yes |
| **Hopkinson et al.**  **2024 *2*** | 3D printed targets and ovine and unstained human tissue | TCSPC enabled SPAD array | Yes | Yes |
| **Hall et al. *3*** | Turbid liquid medium | Photomultiplier tube coupled to TCSPC system | No | Yes |
| **Han et al *4*** | Fluorescent inclusions in phantom and *in vivo* mouse | TCPSC enabled SPAD | Yes, but via scanning optics | Yes |
| **Smith et al *5*** | Fluorescent inclusions in a liquid phantom | Gated-ICCD | Yes | Yes |
| **Zhao et al *6*** | Vegetation autofluorescence | Avalanche photo diode | No | Yes |
| **Bruza et al *7*** | Fluorescent inclusions in a phantom and tumour tissue | Time gated SPAD array | No | Yes |
| **Petusseau et al *8*** | Fluorescent inclusions in phantoms (including tissue-like phantom) and tumour tissue | Time gated SPAD array | No | Yes |

1 – Hopkinson et al, BMOE, 2023

2 - Hopkinson et al, SPIE, 2024

3 - Hall et al, Optics Letters, 2004

4 – Han et al, Biophysical Journal, 2010

5 – Smith et al, Optics Letters, 2020

6 – Zhao et al, Sensors, 2020

7 - Bruza et al, Optica, 2021

8 – Petusseau et al, Journal of Biomedical Optics, 2024

# Figure S1 – Time gating options

#
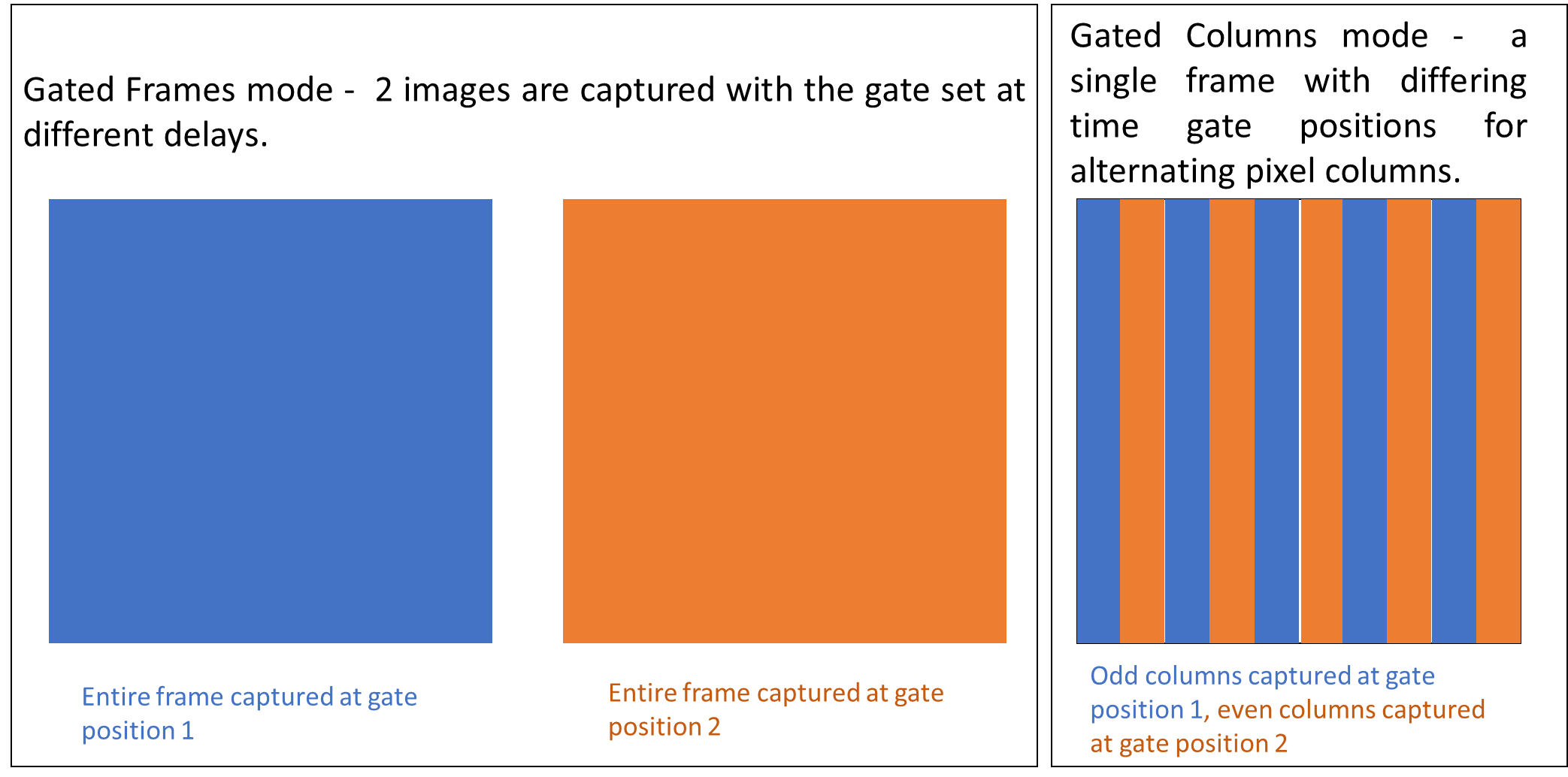


Figure 1 – Schematic to illustrate the two different gating modes available for RLD gating. The two different gate positions are represented with the different colours. In the gated frames mode (left), two separate frames are captured with the entire frame receiving each gate successively. In the gated columns mode (right), odd columns receive one gate, while even columns receive a different gate. This allows lifetime to be calculated from a single frame acquisition, but at the expense of spatial resolution.

# Figure S2 – RLD diagram


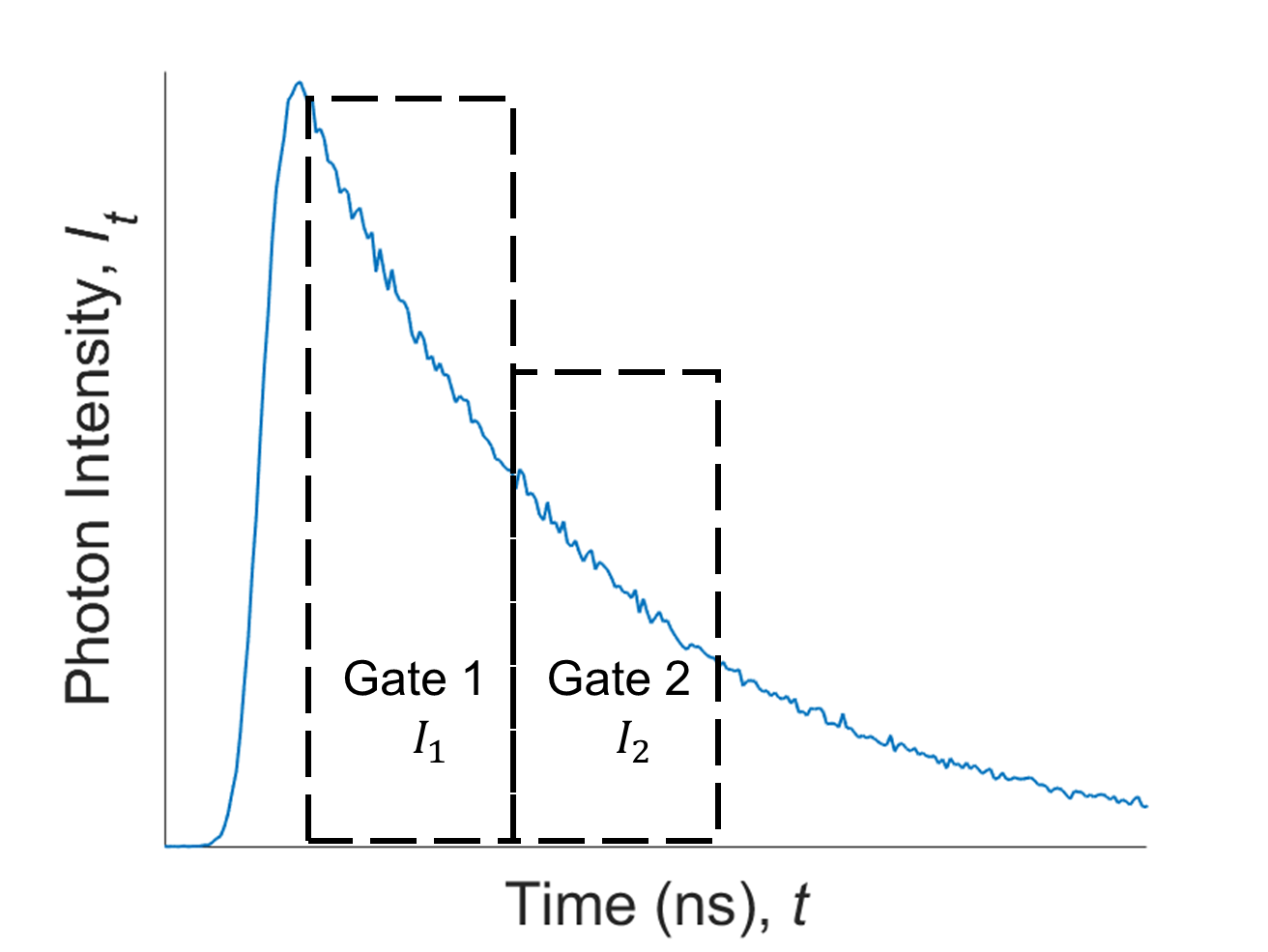


Figure 2 - Schematic to show the placement of the time bins in an RLD measurement.

# Figure S3 – Highlight of motion lifetime artefact


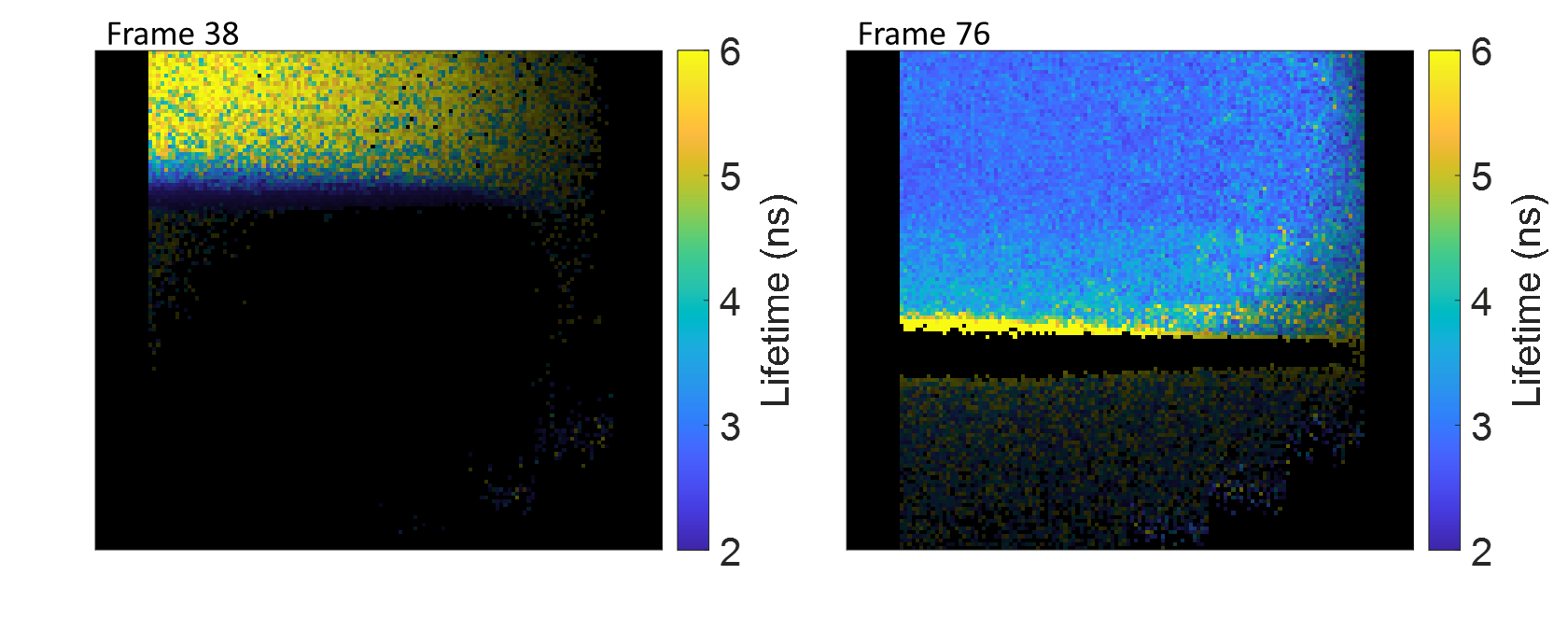


Figure 3 – Stills taken from Video 1 to highlight the artefact of lifetimes on the edges of moving objects being erroneously calculated when using alternating frame gating. Note the bottom edge of the object of Frame 38 has an anomalously short lifetime in this frame, while the edge of the object in Frame 76 has an anomalously long lifetime.

# Figure S4 – Time offset correction


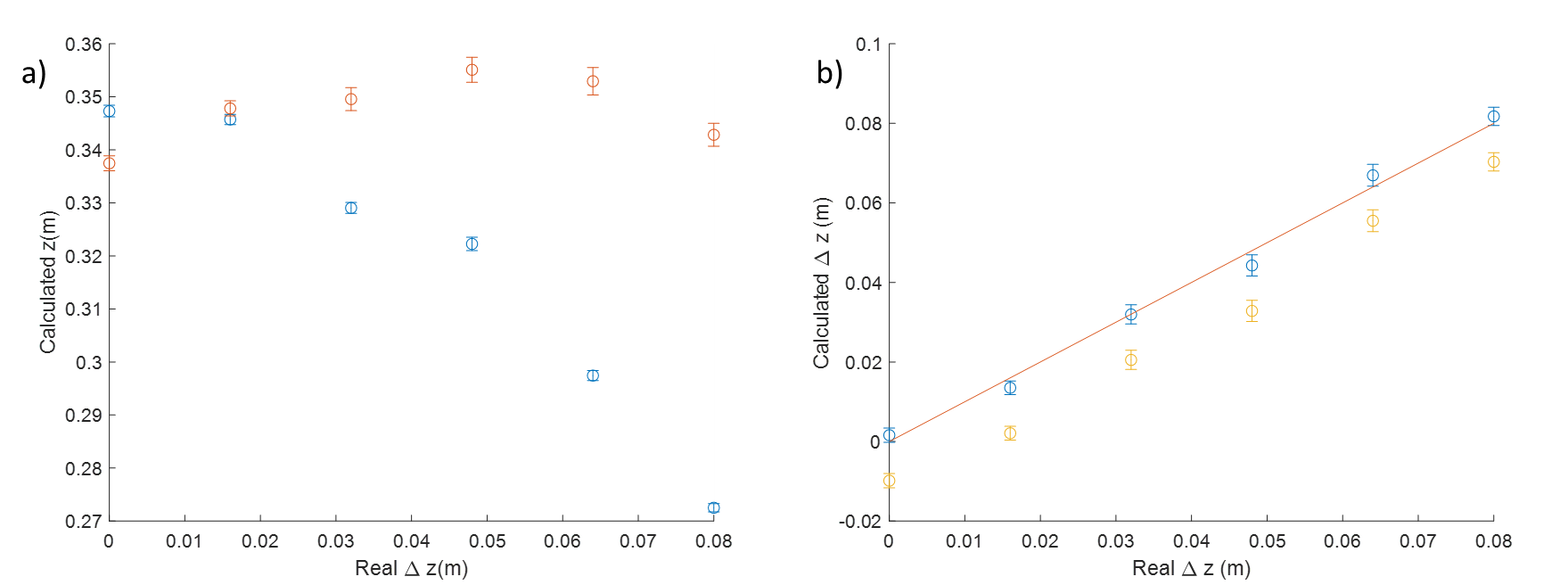


Figure 4 - a) the raw positions calculated for the left hand and right hand object. Notice that although the same distance from the camera, the left hand, stationary object is measured to be ~ 10 mm closer. b) Object separation, showing the uncorrected object separation (yellow) and the corrected separation (blue), along with the real data red. By adding a 11 mm linear offset to the uncorrected yellow data, the blue symbols follow the red line very closely.

# Figure S5 – Highlight of ovine tissue

#
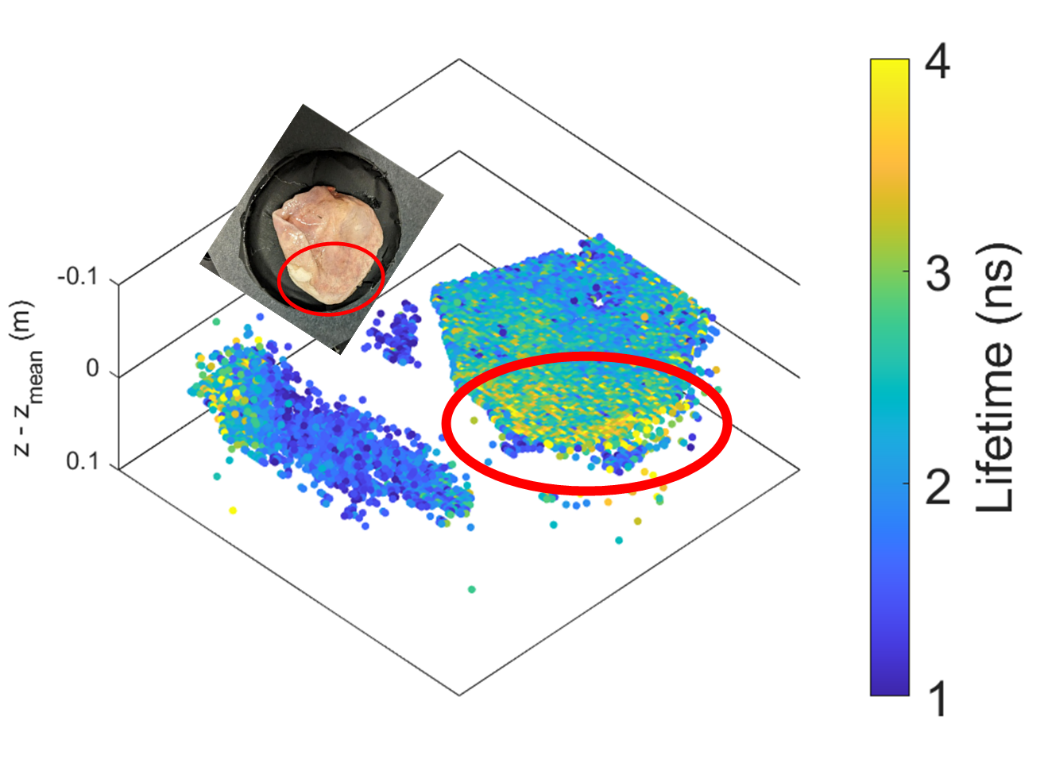


Figure 5 – Figure 6a) with the fatty tissue at the edge of the larger sample highlighted by a red oval, inset is a photograph of the same sample with the same region approximately highlighted.
